# Population genetics analysis of *Diospyrosmun* A.Chev. ex Lecomte (Ebenaceae) based on EST-SSR markers derived from a novel transcriptome

**DOI:** 10.3897/BDJ.12.e130385

**Published:** 2024-09-18

**Authors:** Xuan Thi Tuyet Bui, Duy Dinh Vu

**Affiliations:** 1 Institute of Ecology and Biological Resources, Vietnam Academy of Science and Technology, Hanoi, Vietnam Institute of Ecology and Biological Resources, Vietnam Academy of Science and Technology Hanoi Vietnam; 2 Join Vietnam–Russia Tropical Science and Technology Research Center, Hanoi, Vietnam Join Vietnam–Russia Tropical Science and Technology Research Center Hanoi Vietnam

**Keywords:** *Diospyrosmun*, genetic diversity, endangered species, SSR markers, transcriptome

## Abstract

*Diospyrosmun* A.Chev. ex Lecomte (Ebenaceae), a native evergreen tree in Vietnam, has important economic and ecological values. The absence of effective and reliable molecular markers has hampered the study of *D.mun’s* genetic diversity and population structure, even though it is an endemic and endangered species. Therefore, significant enrichment of genomic resources is urgently needed to uncover and better understand the genetic architecture of *D.mun*. This study aims to demonstrate an efficient and reliable tool to explore the polymorphism within *D.mun* germplasm. It provides a valuable platform for the breeding and conservation of this species and other endangered species worldwide. The Illumina HiSeq™ 4000 sequencing technology was applied for the transcriptomic analysis, genetic differentiation and population structure of *D.mun* in Vietnam. In this study, the transcriptomes of *D.mun* were analysed using the Illumina HiSeq^TM^ 4000 sequencing system and a total of 5,588,615,700 base pairs were generated. *De novo* assembly indicated that 91,134 unigenes were generated (average length = 645.55 bp, N50 = 957 bp, Q20 = 98.08% and Q30 = 94.51%). A total of 92,798 and 21,134 unigenes had significant similarities amongst Nr and Swiss-Prot, respectively. In the GO database, 19,929 unigenes were annotated and these genes were divided into three major categories and 50 subcategories. In the KOG analysis, 18,499 unigenes were annotated and divided into 25 gene function categories. In the KEGG analysis, 12,017 unigenes were annotated. According to the related pathways involved, they could be classified into 56 subclasses. In this study, we have identified a total of 9,391 EST-SSR markers. Ten microsatellite loci were employed to assess the genetic diversity and structure of 82 adult *D.mun* trees across three populations in Vietnam. The results indicated moderate levels of genetic diversity with *PIC* = 0.77, *N_A_* = 3.9, *N_E_* = 2.8, *Ho* = 0.56 and *H_E_* = 0.58 and the fixation index value was recorded as positive for three populations (NS, NH and CP). Genetic differentiation among populations was low (F_ST_ = 0.045), suggesting limited gene flow (Nm = 5.34). This result indicates gene exchange between the populations of ancient *D.mun* from different geographical areas and regions. The analysis of molecular variance (AMOVA) showed that high genetic variation existed within individuals (91%) compared to amongst populations (4%). Genetic structure analysis, DAPC and the NJ tree indicated that the three populations were divided into three main clusters. With this study, we provide a molecular resoureces for the breeding and conservation of *D.mun*.

## Introduction

*Diospyrosmun* A.Chev. ex Lecomte (Ebenaceae) is an endemic and rare species from Vietnam, this species only growing in restricted habitat at higher altitudes in Tuyen Quang, Hoa Binh, Ninh Binh, Ninh Thuan and Binh Thuan Provinces (Nguyen 2003, Ngo 2014, Vu et al. 2014). They have been extensively used for furniture, handicrafts, construction the commercial trade as well as a source of valuable medicine or ornamental plants. Globally, *D.mun* is listed as Critically Endangered under criteria A1cd (Nguyen 1998). In Vietnam, due to the reduction of habitat and over-exploitation, all these species have been listed as Endangered (EN A1c,d, B1 + 2a) (MOST and VAST 2007). Currently, questions on the genetic analyses of this species remain unanswered due to the limitation of available molecular markers. Therefore, it is necessary to conduct research into the fields of distribution, ecology, natural active components, phytochemicals and any other potential resources that *D.mun* possesses to ensure its survival. Accordingly, the data from the transcriptome sequence were utilised to enhance the effectiveness of the SSR generation process for the species.

Conservation and management of a species require information on the ecological and genetic diversity within and amongst populations (Vu et al. 2016, Liu et al. 2023, Yan et al. 2024). To gather th data, particularly to gain deeper insights into genetic processes, advanced biological techniques are necessary (Durmaz et al. 2015). Genetic diversity is reflected in the wide variation of phenotypes and genotypes in plants (Govindaraj et al. 2015). Polymorphic genes lead to the presence of heterozygous genotypes within populations (Li et al. 2023). This variation in genotypes allows populations to adapt to environmental changes and interact with other populations. Furthermore, genetic diversity serves as the foundation for heterosis development (Salgotra and Chauhan 2023).

To some extent, if genetic diversity between parents is greater, the higher the heterosis. Populations with high genetic diversity are valuable as breeding material for genetic improvement programs (Pham et al. 2024). These are factors that help the organism's genetic lineage, resistance to epidemics and adaptation to the changes in environmental conditions. Assessment of genetic diversity may rely on morphological characteristics, agronomy and the genetic level of the plant. Methods of evaluation are based on morphological characteristics and agronomy is conducted easily, but mistakes can be made because phenotypic expressions are the interactions between the genotype and the environment or are due to subjective assessment by researchers.

The emergence of next-generation sequencing technology in recent years, particularly high throughput transcriptome sequencing, has offered extensive expressed sequence data for plants that are not models (Egan et al. 2012, Vlk and Řepkova 2017). Transcriptome sequencing is a simple and effective tool used to develop a large number of unigene-based microsatellites (Simple sequence repeats, SSR) for comprehensive analysis of genome in plants (Vu et al. 2020, Zhang et al. 2021). Transcriptome sequences generally represent the genetic information of gene expression and may be directly related to gene function (Vu et al. 2020, Li et al. 2023). Recently, the development of SSR markers has been reported in persimmon - *Diospyroskaki* Thunb. (Soriano et al. 2006, Naval et al. 2010, Guo and Luo 2011, Luo et al. 2013, Liang et al. 2015, Wang et al. 2018, Li et al. 2019, Wang et al. 2020, Samarina et al. 2021, Li et al. 2023), date-plum (*Diospyroslotus* L.), Chekiang persimmon (*Diospyrosglaucifolia* Metc.) and oily persimmon (*Diospyrosoleifera* Cheng.), Jinzaoshi (*Diospyros* sp.) (Yang et al. 2015), but transcriptome sequences have not been reported in the development of SSR markers in *D.mun*. Moreover, many ESTs for different plant species have been available in recent decades. However, the genetic diversity and genetic structure of *D.mun* have not been explored, which are important prerequisites for formulating and implementing conservation and restoration measures. Therefore, transcriptome sequencing needs to be conducted to understand the genetic and metabolic adaptations in its habitats. An examination of the genetic diversity present within each population and between them is necessary to enhance conservation and management efforts about this species. As a result, this will contribute to improved knowledge of ecology, evolution and conservation genetics for this species. A transcriptome is the complete set of all RNA molecules that includes mRNA, tRNA, rRNA and other non-coding RNA in one cell or a group of cells in a particular environment or a developmental stage of the species.

The Illumina HiSeq^TM^4000 platform was used in this study to obtain characteristics of the comprehensive transcriptome of *D.mun* and to develop a large number of expressed sequence tags - SSR (EST-SSR) markers. The genetic structure of all of the individuals studied, which came from three different populations, was analysed with the help of these markers and the degree of genetic variability within each population and between the populations was calculated. Following this, the study's findings are incorporated into this species' management decisions, conservation efforts and restoration efforts.

## Materials and Methods

### Sample collection and RNA extraction

**Figure 1.**
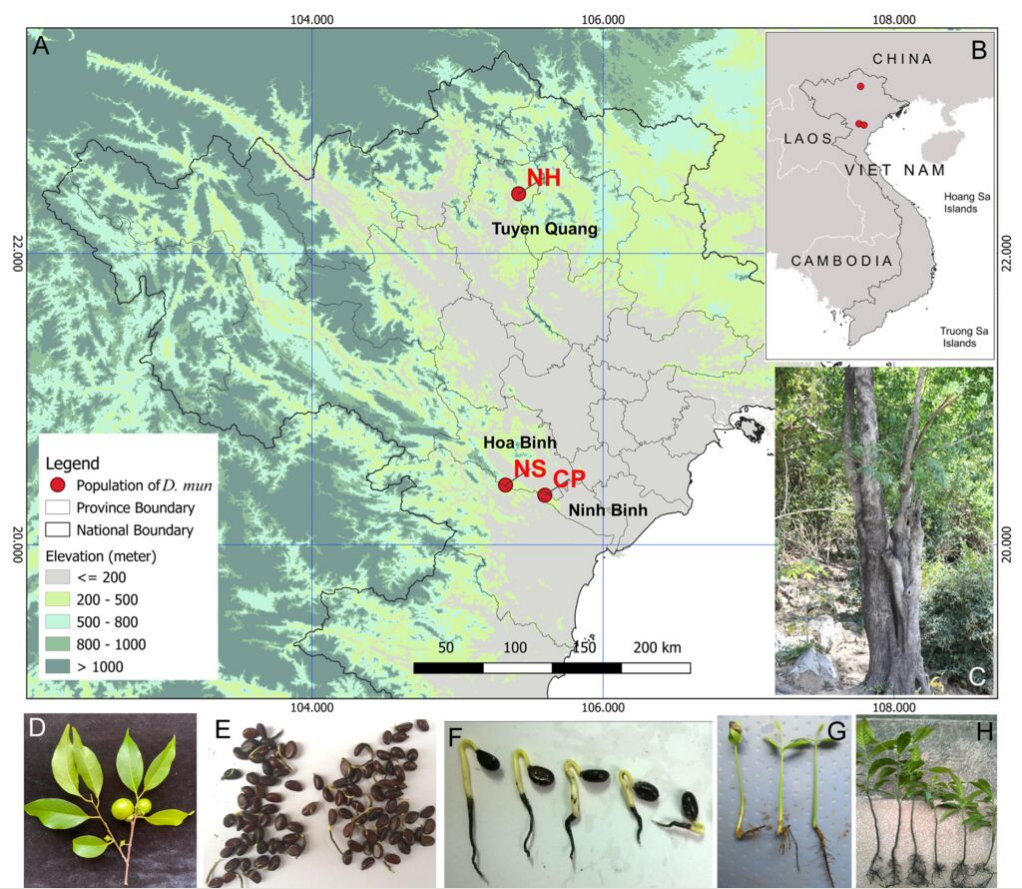
Map of field survey locations and geographic distributions of *D.mun* in the study. Map showing the collection sites (**A, B**), leaves and fruits (**D**), adult plant (**C**), seeds and sprouted seeds (**E, F, G**) and seedling (**H**).

The leaves and stems of *D.mun* were collected in Ngoc Son Nature Reserve, Hoa Binh Province in May 2024 (Fig. 1) and immediately placed in liquid nitrogen in the field. They were later transported to the Laboratory of Molecular Biology, Institute of Ecology and Biological Resource, where they were stored at -80^o^C for RNA extraction. To perform Illumina sequencing, total RNA was first extracted from each sample using a plant RNA Kit and then processed with DNase I. These procedures followed the manufacturer's instructions (Omega Biotack, Inc.). The quality of the processed RNA samples was checked by running electrophoresis on 1% agarose gels and Agilent 2100 Bioanalyzer (Agilent Technologies, CA, USA) and the quantity was checked using Nanodrop ND-2000 spectrophotometer (NanoDrop Technologies, DE, USA). Finally, equal amounts of RNA from different samples were pooled for RNA sequencing.

### Illumina sequencing and de novo transcriptome assembly

RNA samples were sent to Breeding Biotechnologies Co., Ltd. for mRNA-seq library construction and sequencing using Illumina HiSeq^TM^ 4000 next-generation platform technology (Illumina, InC., CA, USA). The quality of the raw reads was checked. The adapter contaminants with the raw reads > 5% of unknown nucleotide (N) and > 20% low-quality bases (quality value < 10) were removed to obtain high-quality clean reads. The clean reads were de novo assembled to construct contigs using TRINITY (Grabherr et al. 2011). The contigs were clustered into unigenes using TGICL v.2.1 (Pertea et al. 2003).

### Functional annotations

To determine the predicted function, all unigene transcripts were used and compared to the NCBI non-redundant (Nr) protein database (Deng et al. 2006) and Swiss-Prot database (Lussi et al. 2023), Gene Ontology (GO) (Hinderer and Moseley 2020), eukaryotic Orthologous Groups of proteins (KOG) (Koonin et al. 2004) and Kyoto Encyclopaedia of Genes and Genomes (KEGG) (Kanehisa 2004) databases using BLAST (E-value of 10^-5^) to search for homologs. Sequences were aligned with the Protein family database (Pfam) to obtain unigene annotation information using the HMMER (Finn et al. 2013). BLAST2GO software (Conesa et al. 2005) was used to determine gene ontology (GO) annotations of assembled unigenes for the categories of biological processes and molecular functions of cellular components. The BLAST software aligned the unigene sequences to the eukaryotic Orthologous Groups (KOG) clusters. KEGG pathway annotations were analysed for the metabolic pathways and the related gene functions.

### DNA extraction, microsatellite polymorphism identification

First, we considered 5,486 SSR designed loci, amongst the 100 SSR primer pairs randomly selected for PCR identification and 10 were successfully amplified by 82 *D.mun* individuals from three natural populations (Fig. 1, Table 1). The total genomic DNA was extracted from the plant DNA Kit according to the manufacturer's instructions (BioTeke, Beijing, China). The DNA purity and integrity were tested by Nanodrop ND-2000 spectrophotometer (NanoDrop Technologies, DE, USA) and then diluted to 20 ng‧µl^-1^. The PCR amplification reaction was performed in a 25 µl reaction volume, comprising 2.5 µl of template DNA, 12.5 µl of 2X Taq Master mix, 1 µl of each primer and 8 µl deionised water. The GeneAmp PCR System 9700 (Applied Biosystems, USA) was used for the PCR amplification with the following conditions: initial denaturation at 94°C for 3 minutes, 40 cycles at 94°C for 30 s, a primer-specific annealing temperature for 30 s and 70°C for 1 min; followed by a final extension at 72°C for 10 min; then maintaining the samples at 4°C until they were analysed. The PCR products were sized and subjected to relative quantification between samples on a 5300 Fragment Analyzer (Agilent) with an Agilent DNF-905 dsDNA Kit (1-500 bp) (Agilent).

### Data analysis

**Genetic diversity**: Null alleles and other genotyping errors were detected using the MICRO-CHECKER v.2.0 software (Van Oosterhot et al. 2004), with 1000 bootstrap iterations over loci to generate the expected homozygote and heterozygote frequencies. CERCUS (Kalonowski et al. 2007) was used to estimate each locus' *PIC* (polymorphism information content) value. Variables for genetic diversity per loci and population, including the number of alleles (*N_A_*), number of effective alleles (*N_E_*), percentage of polymorphic loci (P%), the observed heterozygosities (*Ho*), the expected heterozygosities (*H_E_*), inbreeding coefficient (*F_IS_*) and gene flow (*Nm*) were calculated using the programme GenAlEx v.6.5 (Peakall and Smouse 2012). The individual inbreeding model was performed to evaluate the *F_IS_* index for null allele frequency (*F_IS_IIM*) using INEst (Chybicki and Burczyk 2008). Deviations from Hardy-Weinberg quilibrium (HWE) for loci within populations were tested in CERCUS, based on 1,000 permutations of alleles amongst individuals.

**Genetic differentiation and population structure**: The *F_ST_* (Weir and Cockerham 1984) and *G'_ST_* values (Hedrick 2005) were able to quantify the genetic divergence that exists between the populations by GenAlEx v.6.5. Using ARLEQUIN v.3.1, the significance of the *F_ST_* values in population pairs was assessed at a significance level of 0.05. The results of this testing were considered significant (Excoffier et al. 2017). The analysis of molecular variance (AMOVA) was carried out with the assistance of ARLEQUIN v.3.1, which was used to carry out the significance testing for the variance components involved. The outcomes of 10,000 different permutations served as the basis for this testing. To analyse the population structure using STRUCTURE v.2.3.4, the Bayesian clustering method was used (Pritchard et al. 2000). Establishing the admixture model with correlated allele frequencies required ten distinct runs for each of the groups in the dataset (K). These runs were carried out with 500,000 Markov Chain Monte Carlo (MCMC) iterations and a burn-in time of 100,000 iterations. K ranged from 1 to 15. STRUCTURE HARVESTER (Earl and von Holdt 2011) was used to detect the number of groups that best fit the dataset, based on the K that was determined by Evano et al. (2005) to determine the ideal value of K. This was done so that the optimal value of K could be determined. When the optimal K value had been determined, the duplicated findings were aligned with the help of CLUMPP v.1.1.2 (Jakobsson and Rosenberg 2007) and the allocated cluster membership bar plots were created with DISTRUCT v.1.1. (Rosenberg 2003). A Discriminant Analysis of Principal Components (DAPC) was also carried out by utilising the Adegenet package that was available for the R v.4.0.2 programme to discover clusters of individuals with a common genetic ancestor (Jombart et al. 2010). The DAPC was implemented without any previous information on the population's origin. The Bayesian Information Criterion was used to establish the "ideal" cluster size and the number of clusters, denoted by the letter "K," ranged from one to twenty in the experiments (BIC). The DAPC was also responsible for performing the previous information analysis to determine how individuals should be assigned to populations. To obtain a better visual representation of the genetic clusters, the complot function of adegenet was included. The *xval*DAPC function was used to keep the top fourteen principal components of the principal component analysis (98.5% of the variance that was conserved), as well as the seven discriminant eigenvalues. The tree topologies of all individuals were constructed, based on the Neighbour-Joining (NJ) method using NTSYS v.2.11 (Saitou and Nei 1987) and MEGA v.11.0 (Tamura et al. 2021).

## Data resources

All data generated or analysed during this study are included in this published article [and its supplementary information ﬁles]. Raw data, including biological replicates, are available from the corresponding author on reasonable request.

## Results

### Illumina sequencing and de novo assembly

Through RNA-seq, 18,628,719 original sequences of *D.mun* were obtained with a total of 5,588,615,700 base pairs, Q20 (98.08%) and Q30 (94.51%). The number of ‘N” bases (ambiguous bases) and GC-content were 0% and 46.82%, respectively. It showed that the high-throughput sequencing platform ha obtained a higher quantity and quality of *D.mun* sequences, which is conducive to the subsequent data assembly and meets the needs of later bioinformatics research. After the clean reads were assembled by de novo, a total of 157,553 transcripts with sequence information of 166,642,297 bp and 91,134 unigenes with sequence information of 58,649,736 bp were obtained (Table 2). Analysis of the sequence length of the transcript shows that its average length was 105.77 bp and N50 was 1,867 bp. Amongst them, short sequences of 200 to 300 bp were the majority, with 37,823 sequences accounting for 24% of the total; sequences with a length of 300 to 500 bp were 32,955 (20.92%) sequences; sequences of 500 to 1,000 bp in length were 30,838 (19.57%) sequences; 31,236 (19.83%) sequences ranged from 1,000- 2,000 bp and sequences longer than or equal to 2,000 bp accounted for 24,701 (15.68%) sequences. Unigene analysis and statistical results show that its average length was 645.55 bp N50 was 957 bp, of which the sequences of 200 to 300 bp account for 33,327 (36.57%) of the total sequence; 26,456 (29.03%) sequences were between 300 and 500 bp; 17,051 (18.71%) sequences 500 to 1,000 bp; 9,067 (9.95%) sequences 1001 to 2000 bp and the sequences of more than 2,000 bp account for 5,233 (5.74%) (Table 2). By processing a large number of sequences obtained by high-throughput RNA-seq, the integrity of unigenes data after assembly is significantly improved and the next step of analysis and statistics can be performed.

### Functional annotation and classification of unigenes

A total of 91,134 unigenes were obtained through BLAST software in eight major databases (COG, GO, KEGG, KOG, Pfam, Swissprot, eggNOG and Nr) (Table 3). Amongst them, 32,798 unigenes were successfully annotated in Nr, accounting for 35.99% of the total number of unigenes; in KEGG, there were 12,017 successful annotations, accounting for 13.19% of the total; in SwissProt, there were 21,134 successful annotations, accounting for 23.19% of the total; 20,799 successful annotations in Pfam, accounting for 22.82% of the total; 19,929 successful annotations in GO, accounting for 21.87% of the total; 18,499 successfully annotated in KOG, accounting for 20.30% of the total; 9,666 successfully annotated in COG, accounting for 10.61% of the total and, in eggNOG, there are 30,531 successful annotations, accounting for 33.50% of the total. The number of successfully annotated sequences in all eight major databases was 33,421, accounting for 36.67%.

Through the Nr library's comparison, 32,798 unigenes of *D.mun* found similar sequences in the Nr database (Suppl. material 1). The annotation matching species mainly include *Nelumbonucifera* (6,032; 18.41%), *Macleayacordata* (3,484; 10.63%), *Vitisvinifera* (1,689; 5.15%), *Elaeisguineensis* (1,240; 3.78%), *Phoenixdactylifera* (1,110; 3.39%), *Aquilegiacoerulea* (916; 2.80%), *Quercussuber* (625; 1.91%), *Plicaturopsiscrispa* (531; 1.62%), *Amborellatrichopoda* (524; 1.60%) and *Juglansregia* (510; 1.56%). A large fraction indicated similarities to genes in other species (16,109; 49.16%). From the annotated information, it can be concluded that most of the sequences of *D.mun* can be matched in angiosperms. In general, from the distribution of sequence similarity, it can be seen that *D.mun* has a high matching degree in the Nr database. However, due to the lack of genome and transcriptome information of *D.mun*, no match in the database was found for some unigenes.

**Figure 2.**
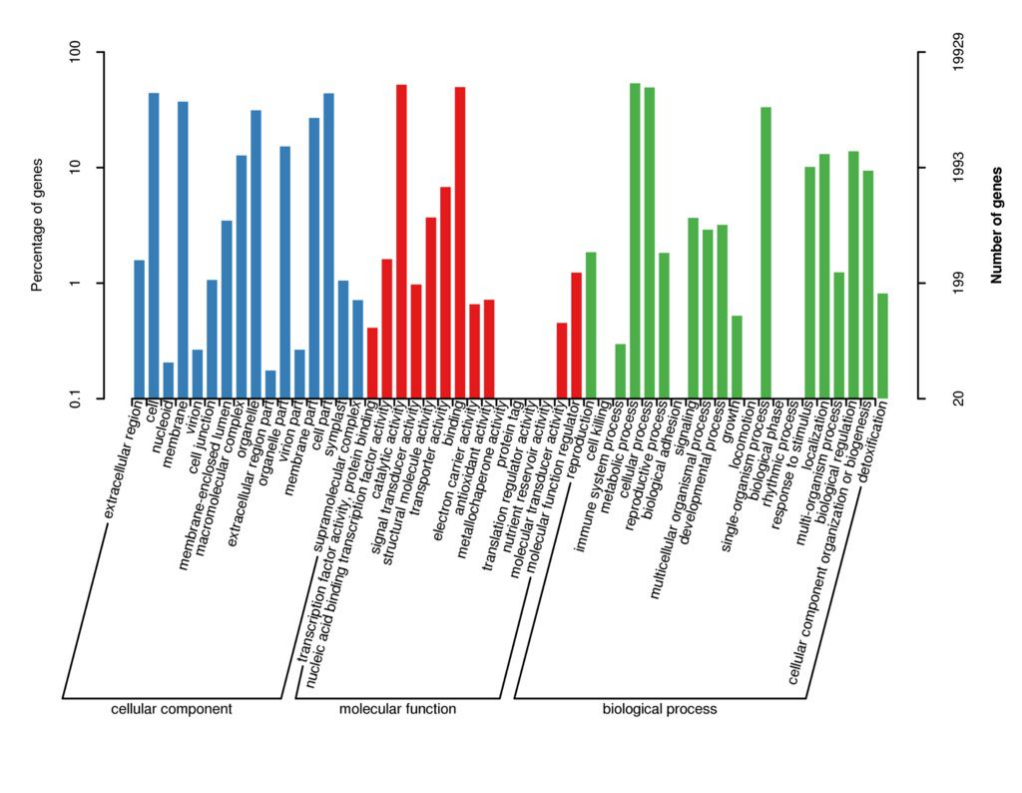
Gene Ontology (GO) classifications of unigenes of *D.mun*. A total of 19,929 GO functions were categorised into three main categories: biological process, cellular component and molecular function. The x-axis indicates the subgroups in GO annotation, while the y-axis indicates the percentage of speciﬁc categories of genes in each main category.

According to the genes successfully annotated by Nr, the GO function classification annotation was performed and the result is shown in Fig. 2. The analysis results show that 91,134 unigenes have annotated 19,929 GO functions, accounting for 21.87% of the total number of unigenes. Divided into three major functional categories, there are 43,996 gene sequences of cellular component functional categories, accounting for 40.98% of the total; 39,750 gene sequences of biological process functional categories, accounting for 37.02% of the total and 23,617 gene sequences of molecular functional categories, accounting for 22% of the total. It can be observed that the proportion of genes annotated in the functional category of a cellular component is the largest. The three functional categories can be further divided into 52 GO functional subcategories, including 16 cellular components, 15 molecular functions and 21 biological processes. Amongst the 21 functional subcategories in biological processes metabolic processes (1,0697; 26.91%), cellular processes (9,859; 24.8%) and single-organism processes (6,642; 16.71%) have received more annotations. The percentage of annotations obtained during the biological phase in biological processes was the lowest, having only (2; 0.005%). In the category of cellular components, most of the annotations for cell (8,832; 20.07%), cell part (8,784; 19.97%), membrane (7,440; 16.91%) and organelle (6,218; 14.13%) have accounted for the extracellular region part (35; 0.08%) and nucleoid (41; 20.07%) were less annotated. In the molecular function category, there are many annotations for catalytic activity (10,393) and binding (9,895), each accounting for 44% and 41.9% of the total classification, while the annotations for metallochaperone activity (2), translation regulator activity (3) and protein tag (4) account for at least 0.008%, 0.013% and 0.017%, respectively. The above GO function annotation results show the basic situation of gene expression in *D.mun*. It can be seen that amongst the three functional subcategories, there are more genes related to metabolic activities in biological processes, indicating that *D.mun* has a strong metabolic ability.

**Figure 3.**
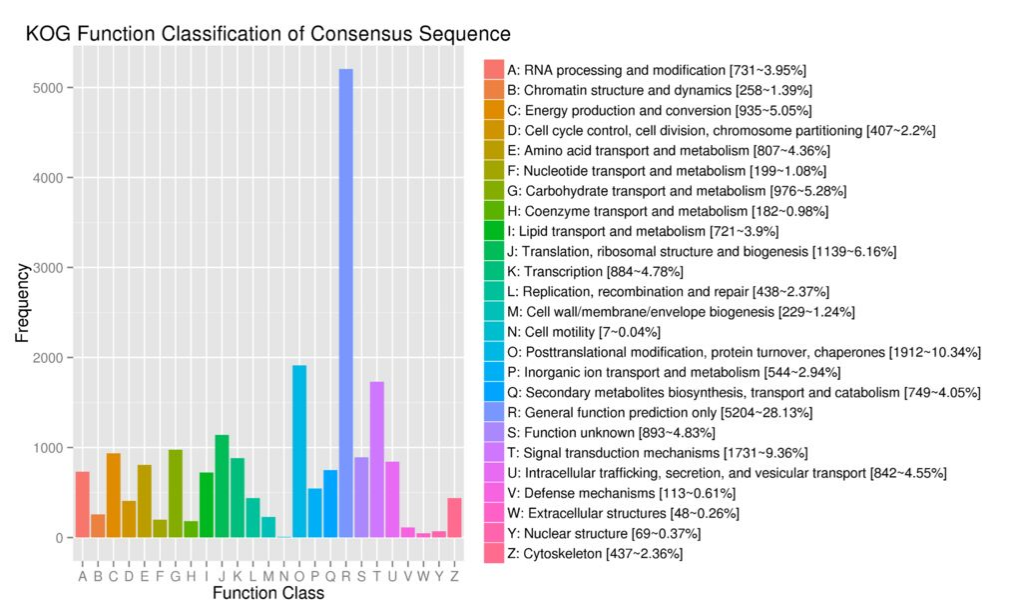
Eukaryotic Orthologous Groups (KOG) classification of unigenes. Functional predictions of unigenes were classified into at least 25 function classes according to the KOG database.

The obtained unigene is classified and annotated by the KOG protein database and the results are shown in Fig. 3. The analysis results show that 18,499 unigenes in KOG can be matched, accounting for 20.30% of the total. The comparison results can be divided into 25 functional categories, including energy production and conversion, translation, ribosomal structure and biogenesis and different types of gene expression, such as biosynthesis, processing and secondary metabolites biosynthesis, transport and catabolism. "General function prediction only" was the primary group (5,204; 28.13%), followed by "post-translational modification, protein turnover and chaperones" (1,912; 10.34%), "signal transduction mechanisms" (1,731; 9.36%) and "translation, ribosomal structure and biogenesis" (1,139; 6.16%). The cell motility (7; 0.04%) and extracellular structures (48; 0.26%) have the least functional annotation information. *D.mun* has more gene expression in terms of transcription, translation and protein transport. In addition, there is one function unknown (893; 4.83%), whose specific biological function cannot be known.

**Figure 4.**
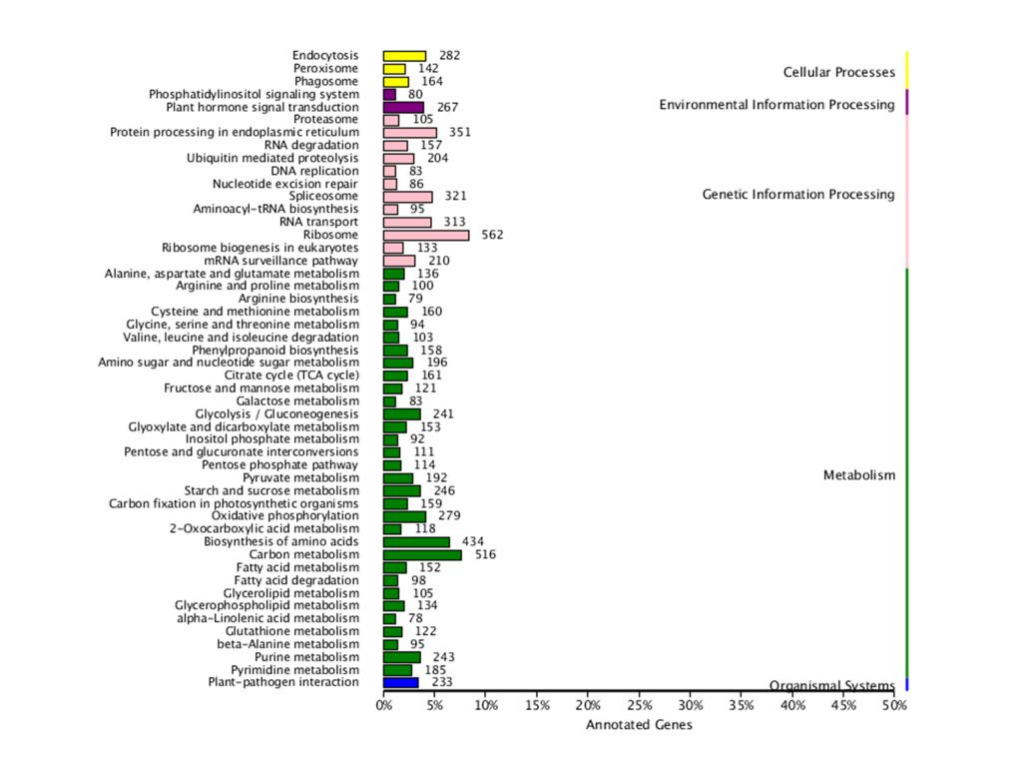
Kyoto Encyclopaedia of Genes and Genomes (KEGG) metabolism pathway classification of unigenes. Data were classified into five main categories: metabolism, genetic information processing, environmental information processing, cellular processes and organismal systems. Bars represent the number of *D.mun* unigene matches in each category.

Comparing the obtained unigenes to the KEGG database, 12,017 unigenes were annotated, accounting for 13.19% of the total. According to the metabolic pathways involved, *D.mun* can be classified into five major categories (Cellular Processes, Environmental Information Processing, Genetic Information Processing, Metabolism, Organismal Systems) and 50 subcategories (Fig. 4). Through the specific analysis and statistics of unigenes under the relevant pathway classification in Fig. 4, it was found that the metabolism pathways account for the largest proportion of the five categories, with 5,258 unigenes, followed by pathways related to genetic information processing (2,620 unigenes), cellular processes (588 unigenes) and environmental information processing (347 unigenes). Pathways related to organic systems (233) were the fewest. The five major categories were further subdivided into subcategories. Amongst them, metabolism-related pathways were divided into 32 subcategories. In addition, pathways related to genetic information processing were divided into 12 subcategories. In the environmental information processing pathways, there are only two subcategories. In the cellular processes, there were three subcategories. Pathways related to organic systems had only one subcategory. In the KEGG metabolic pathway analysis results, the category with the most annotated genes indicated that *D.mun* has strong metabolic activities.

### Frequency and distribution of SSRs in the unigenes

All 14,300 assembled unigenes with 28,647,926 bp were examined to discover potential EST-SSRs (Suppl. material 3). A total of 9,391 SSRs were found amongst 91,134 unigenes. Of the 9,391 SSRs, 6,545 SSR-containing sequences were found, as were 2,035 sequences with more than one microsatellite locus and the number of SSRs involved in compound formation (586).

The frequency of microsatellites in the *D.mun* unigenes was 14.33%. Of the 9391 potential SSRs, distribution to different repeat type classes was identified (Suppl. material 2): mononucleotide and dinucleotide repeats were the most abundant, with 5,030 (53.56%) and 2,384 (25.39%), respectively, followed by trinucleotide repeats (1,865; 19.86%), tetranucleotide repeats (85; 0.91%), pentanucleotide repeats (17; 0.18%) and hexanucleotide repeats (10; 0.11%). It can be seen that the main repeat types of microsatellite sites in *the D.mun* transcriptome are tri-nucleotide repeats and dinucleotide repeats.

Regarding the number of repeat units, the distributions and frequencies of nucleotide repeats are presented in Table 4. The microsatellites that contained ten repeat units were the most prevalent (1,735; 18.48%), followed by those that contained six tandem repeats (1,184; 12.61%) and five tandem repeats (1,150; 12.25%), while the remaining tandem repeats contributed less than 10% of the total SSR.

Statistical analysis of EST-SSR, based on motif types in *D.mun* transcriptome was undertaken (Suppl. material 4). In the mono-nucleotide repeats, A/T had a large proportion (4,981; 53.04%). In the di-nucleotide, the dominant nucleotide repeats were AG/CT (1,852; 19.72%). In the tri-nucleotide, AAG/CTT was the most common (720; 7.67%). In the tetra-nucleotide, AAAT/ATTT was the most common (22; 0.23%). Additionally, in the pentanucleotide and hexanucleotide repeats, the types of repeat motifs were evenly distributed, which related to the small number of corresponding microsatellite sites.

### Genetic diversity

Using 10 different polymorphic EST-SSRs, a study was conducted on the standard genetic diversity and the population genetic structure of three different *D.mun* populations (Table 5). Null allele frequencies were not determined at ten loci for *D.mun* at the significant level of 0.05. From the results, Table 5 shows that *N_A_* = 3.96, *N_E_* = 2.67, *PIC* = 0.77, *Ho* = 0.56 and *H_E_* = 0.57. The *F_IS_* value was 0.02. Positive Fis values were found across all four loci under investigation, suggesting an excessive number of homozygotes and inbreeding. It may be deduced that the inbreeding coefficient of the populations (*F*_*IT*_ = 0.06) suggests an excess of homozygosity in the populations.

The number of polymorphic loci varied between populations for *D.mun* species. The percentage of polymorphic loci was high (100%) in all *D.mun* populations (Table 1). Alleles per locus (*N_A_*) averaged 3.9, ranging from 3.8 in the NH population to 4.1 in the CP population for *D.mun*. The lowest value of effective alleles (Ne) was observed in the NH population (*N_E_* = 2.3), whereas this value was the highest in the CP population (*N*_*E*_ = 2.9, *Ho* = 0.57 and *H_E_* = 0.6). The lowest values of *Ho* = 0.53 and *H_E_* = 0.55 were detected in the NH population. The *F_IS_* = 0.029. Significantly positive F_IS_ values were observed in three populations, showing a deficiency of heterozygotes. The *F_IS_IIM* values varied from 0.01 (CP) to 0.03 (NS), with an average of 0.02 (Table 1).

### Genetic structure and cluster analysis

The analysis of molecular variance (AMOVA) was implemented, based on 9,999 permutations and revealed the molecular variation attributable to differentiation amongst and within the populations for *D.mun* (Table 6). The AMOVA showed that 4% of the total genetic variation occurred amongst populations and a significant amount (91%, p < 0.001) of the total variation occurred within populations. This finding was confirmed by the small overall *F_ST_* and large overall *Nm*, which were 0.045 and 5.34, respectively. The *F_ST_* values amongst populations varied from 0.009 (NS and CP) to 0.082 (CP and NH). Significant differentiation was observed for pairwise *F_ST_* values for both the *D.mun* species (p <0.01 and 0.001) (Suppl. material 5).

**Figure 5.**
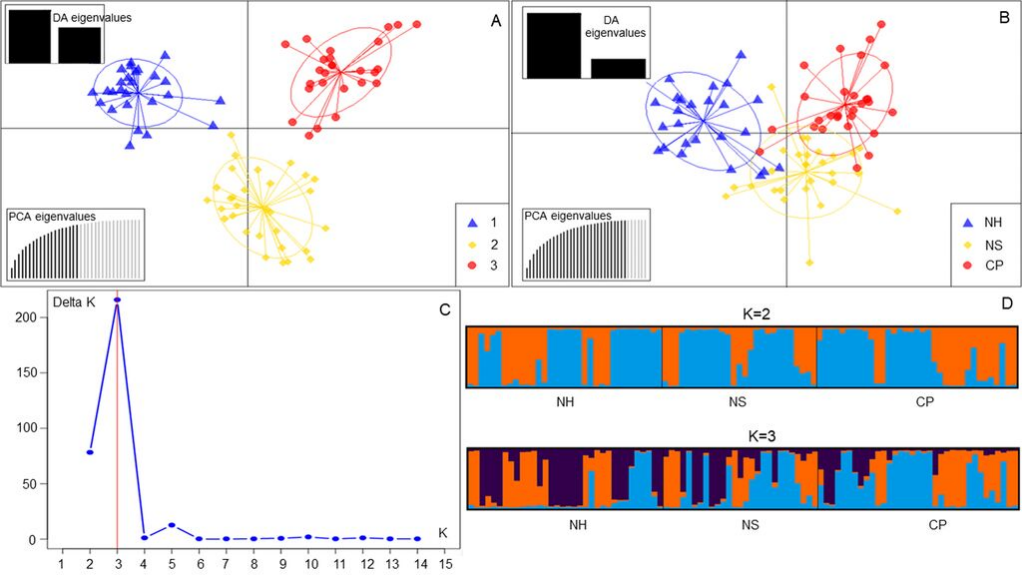
Analysis of population structure using DAPC and STRUCTURE for three *D.mun* populations. Scatterplot of the DAPC with prior information **(A)**, Scatterplot of the DAPC without prior information **(B)**, Distribution of DeltaK over K = 1-15 **(C)** and Bar plots at K = 2 and K = 3 **(D)**.

Without any prior information, discriminant analysis of principal components (DAPC) also uncovered three genetic groupings for *D.mun* (Fig. 5A). Cluster 1 included 14 individuals from the NS population, nine individuals from the NH population and seven individuals from the CP population (Suppl. material 6). Most individuals from the two populations of NH (11 individuals) and CP (14 individuals) were assigned to cluster 2. In cluster 2, there was also one individual in NS. Cluster 3 included most individuals in NS (11 individuals), eight in NH and seven in CP. The DAPC, with prior information on population origin, showed individuals within and between populations (Fig. 5B). The high overlap indicated low genetic differentiation between populations.

The Bayesian analysis of the assignation of individuals, based on the likelihoods, showed that the highest ∆K value (215.9) for 82 individuals revealed K = 3 to be an optimum number of genetic groups and showed that all individuals exhibited admixture from three groups (Fig. 5C). The proportion of ancestry linked with each genetic group might be deduced from the colour of each individual, as measured by their percentage of the population's total colour (Fig. 5D). One group (orange) was predominant in the NS populations with strong ancestry values 47.3% and two populations (NH and CP) with lower ancestry values, 29.4% and 16%, respectively (Suppl. material 7). In the second group (blue), two populations (PH and HSP) represented the largest group (47.7% and 37.9%, respectively), followed by the NS population (16%). Similarly, in the last group (purple), the three populations (NS, NH and CP) were the same (36.8%, 32.7% and 36.3%, respectively). At K = 2, the *D.mun* populations were divided into two groups.

**Figure 6.**
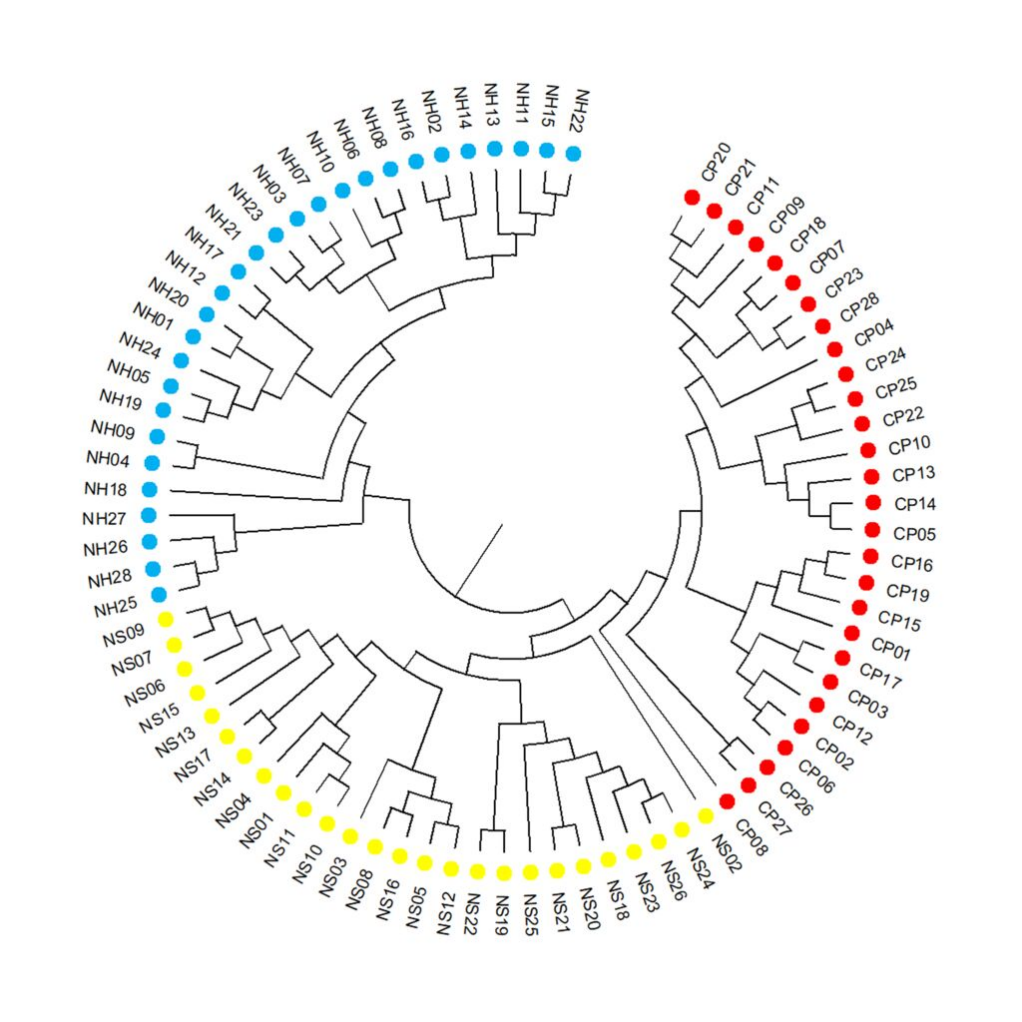
Neighbour-Joining (NJ) tree was constructed, based on Nei’s GDs of 82 genotypes of *D.mun*, based on 10 polymorphic SSR loci.

The NJ tree, based on a matrix of Nei's GDs amongst individuals using NTSYS 2.11 and MEGA 11.0, showed that the relationship of 82 *D.mun* individuals mainly had three branches. The first branch consisted of 28 individuals in the NH population and the second largest branch consisted of 54 individuals (CP and NS populations). The NJ tree was consistent with the STRUCTURE and the DAPC analysis and the relationships amongst many individuals were inconsistent with their geographic location (Fig. 6).

## Discussion

The Illumina Hiseq4000 sequencing platform was used in this study to determine the transcriptome of *D.mun* and bioinformatics techniques to analyse the microsatellite sites in the transcriptome database. The transcriptome sequencing of *D.mun* provided a valuable resource for developing SSR markers to study genetic diversity, molecular marker-assisted breeding and the evolution of the Ebenaceae family. A total of 91,134 transcriptomic unigenes were obtained using the Illumina HiSeq4000 platform for *D.mun*, with a mean length of 645.55 bp and N50 of 957 bp. These lengths are shorter than *Diospyroskaki* Thunb. with N50 = 1,025 bp and average length = 663 bp (Li et al. 2019), indicating that the transcriptome sequencing data were well assembled for *D.mun* in the present study. The methods used in the current study are the same. However, the deviation might be due to the sequencing depth, assembly method and natural characteristics of the species. After assembling the uni-transcripts, the GC content (46.82%) of *D.mun* was lower than that of *D.kaki* (GC = 48.68% (female) and GC = 48.9 (male)) (Li et al. 2019), although the methodology was the same. The GC content provides information regarding the stability of genes and genomic composition for the evolution and genetic structure of the species. These results can be related to species' adaptability to different environmental conditions. The COG, GO, KEGG, KOG, Pfam, Swiss-Prot and NR databases were searched for sequence matches to determine the genetic diversity of *D.mun* and its development. Annotations of GO categories were made on the *D.mun* unigenes. The metabolic process in the biological process and cell in cellular components were the largest groups in this study, indicating important cellular and metabolic activities. These findings are comparable to those found in an earlier investigation by *D.kaki* (Li et al. 2019). A total of 18,499 unigenes were assigned to 25 KOG categories of *D.mun*. The current KOG database results are parallel to those previously reported for 26 in KOG of *D.kaki* (Li et al. 2019). For this purpose, five major categories and 50 subcategories in KEGG resources were used to study genes' biological functions and interactions. As plant hormones play crucial roles in ﬂower sex diﬀerentiation, the plant hormone signal transduction pathway is of particular interest. A total of 267 unigenes were assigned to the plant hormone signal transduction pathway. Additionally, some dominant pathways were ribosome, oxidative phosphorylation, protein processing in the endoplasmic reticulum, glycolysis/gluconeogenesis, spliceosome, RNA transport and purine metabolism. These findings suggest that the *D.mun* unigenes discovered here have broad applicability and will prove useful for future studies of the species' functional diversity.

According to Zalapa et al. (2012), having longer sequences will enhance the likelihood of successfully developing EST-SSR primers, thus *D.mun* was chosen as a prime target for microsatellite analysis in this study. Furthermore, the results indicate 91,134 unigenes in the transcriptome of *D.mun*, which have 8,805 microsatellite loci. At the same time, there are abundant types of microsatellites in the transcriptome of *D.mun*, with six different types of nucleotide repeats, of which the main repeat type is mono-nucleotide repeats (53.57%) and di-nucleotide repeats (25.37%), which are similar to *D.kaki* (Li et al. 2019, Wang et al. 2020). Of these identiﬁed SSRs, the (A/T)n nucleotide repeat was the most abundant.

Improvements to germplasm resources through genetic diversity have been extensively implemented in several other *Diospyros* (Deng et al. 2015, Yang et al. 2015, Wang et al. 2018, Wang et al. 2020, Samarina et al. 2021, Samarina et al. 2022). There is typically a correlation between the number of loci and populations within a species and the level of genetic diversity that exists within that species (Vu et al. 2018), the size of the geographical range that the species inhabits (Nguyen et al. 2021) and genetic exchange (Hellmann et al. 2008). According to the evaluation criteria of polymorphism (PIC > 0.5) (Botstein et al. 1980), the current study found evidence of high genetic variation, based on the EST-SSR (*PIC* = 0.77). Due to the relatively high PIC values, it can be deduced that the chosen loci are both highly informative and appropriate for conducting genetic research on *D.mun*. Several genetic measurements, including the mean number of observed alleles, observed heterozygosity, expected heterozygosity and the percentage of the polymorphic band, corroborate this finding (Liang et al. 2015, Wang et al. 2018, Wang et al. 2020, Samarina et al. 2021). *D.mun* exhibited a moderate degree of genetic diversity (*H_E_* = 0.58; *Ho* = 0.56) compared to other *Diospyros*, such as *D.kaki* (*Ho* = 0.694) and *D.lotus* (*Ho* = 0.2) (Samarina et al. 2021) and *D.kaki* (*Ho* = 0.795) (Wang et al. 2018). Furthermore, our results showed that the observed number of alleles for each locus in *D.mun* (*N_A_* = 3.9) was lower than that compared to *D.kaki* (*N_A_* =11.0) (Samarina et al. 2021). The extensive genetic diversity within a species is a reflection of its evolutionary past and the ecogeographic environment in which it has thrived. It is common for species to maintain a high genetic diversity when they have a wide distribution pattern, a large population size, a long lifespan, a predominance of outcrossing and successional stages (Hamrick and Godt 1996, White et al. 2007). It is hypothesised that species with high genetic heterozygosity will have a greater capacity for adaptability in their environment. This hypothesis is supported by the observation that *Diospyros* species have a broad distribution and live for a very long time. Genetic heterozygosity is related to anthropogenic disturbance and can be reduced through genetic drift and increased homozygosity for common alleles due to the loss of rare alleles (Honnay and Jacquemyn 2007, Gijbels et al. 2015). Genetic heterozygosity in NS, NH and CP was moderate, suggesting that human activity may have contributed (Liu et al. 2023, Yan et al. 2024). In all populations examined, deforestation and overexploitation are potential major factors reducing genetic heterozygosity. Most of the *D.mun* trees with more than 30 cm diameter (dbh) were exploited by forestry enterprises in the 1980s and 1990s (MOST and VAST 2007). The recent populations include trees with age less than 100 years that have fragmented into small populations. The short evolutionary history and tiny population sizes of *D.mun* may account for its moderate genetic diversity. An effective population size and genetic variety decreasing due to inbreeding may have resulted from anthropogenic disturbance and population declines. Significant heterozygosity deficits across 82 trees from three populations suggest that a relatively high heterozygosity deficiency can exist within the *D.mun* populations. This shows that inbreeding exists in small populations, although this species is outcrossed (Murawski et al. 1994, Kenta et al. 2002, Lee et al. 2006).

Gene flow and random mutations contribute to genetic diversity amongst populations (Schaal et al. 2002). Low population genetic differentiation reflects high gene flow. Strong gene flow (Nm > 1) determined a high number of migrants for each generation and may prevent genetic differentiation amongst populations due to genetic drift (Slatkin 1987). According to the findings of our study, a gene flow rate that was comparatively high (Nm = 6.34) had the potential to reduce the impact of genetic drift by lowering the population's level of genetic differentiation, while simultaneously raising the level of genetic heterozygosity. Gene flow is determined via the dispersal of pollen grains and seeds (Finkeldey and Hattemer 2007,Brütting et al. 2012, Budd et al. 2015) and contributes to population genetic differences and genetic structure. *D.mun* are long-lived, predominantly outcrossed, insect-pollinated and late successional (Nguyen 2003, Ngo 2014). Their seeds are dispersed by wind and water (Nguyen 2003). Insects are responsible for pollinating *D.mun*, while wind is responsible for spreading its seeds. The increased isolation distance will result in a reduction in the amount of pollen that is transmitted from population to population of *D.mun*. Pollen transmission is primarily associated with insects. The current population is fragmented into smaller populations. Thus, habitat fragmentation leading to increased isolation can decrease gene exchange and influence its genetic structure in recent decades. The genetic structure of *D.mun* in the present study was also detected via different clustering analyses (Structure, DAPC and NJ), in which 82 studied trees were grouped, based on their geographic origin. Therefore, the genetic structure's existence could result from gene exchange related to geographic distance and habitat fragmentation that separates populations into different genetic groups. Thus, a decrease in the dispersal of pollen and seeds might limit gene flow for *D.mun*.

## Conclusions

The *D.mun* transcriptome was sequenced in its entirety for the first time using the Illumina 4000 sequencing technology. Many ESTs were generated and potentially differently expressed genes in *D.mun* were found. A total of 9,391 EST-SSRs were discovered and 10 microsatellite sites were successful. It is clear from the results that the naturally occurring populations of *D.mun* have maintained a reasonable amount of genetic variation over time. Several SSR markers were found, which will be useful in the future marker-assisted breeding of *D.mun*. We determined a moderate genetic variation and low genetic difference among the three populations of *D.mun*. The structure and the DAPC identified three main genetic groups besides moderate genetic diversity in three populations of *D.mun* species. This research lays the foundation for conservation efforts to protect the species' genetic diversity.

## Supplementary Material

986D9E3B-A6AE-537A-A166-F7294F11604B 10.3897/BDJ.12.e130385.suppl1 Supplementary material 1 Distribution of species search of unigenes against the Nr database. Data type images File: oo_1068814.png https://binary.pensoft.net/file/1068814 Thi Tuyet Xuan Bui, Dinh Duy Vu

6FBB97DA-8973-5749-A464-5F52A702DD6C 10.3897/BDJ.12.e130385.suppl2 Supplementary material 2 Distribution type of EST-SSRs of *D.mun* Data type images File: oo_1068816.png https://binary.pensoft.net/file/1068816 Thi Tuyet Xuan Bui, Dinh Duy Vu

EA4C01C3-0134-5DD8-A570-2F017C2B9982 10.3897/BDJ.12.e130385.suppl3 Supplementary material 3 Summary of analyses of expressed sequence Tag–Simple Sequence repeat (EST-SSRs) in *D.mun* Data type Genomic File: oo_1068861.docx https://binary.pensoft.net/file/1068861 Thi Tuyet Xuan Bui, Dinh Duy Vu

1D02B0E1-4217-537C-9F9E-7A7AD9B80626 10.3897/BDJ.12.e130385.suppl4 Supplementary material 4 Frequency distribution of SSRs based on motif types in *D.mun* transcriptome Data type Genomic File: oo_1068862.docx https://binary.pensoft.net/file/1068862 Thi Tuyet Xuan Bui, Dinh Duy Vu

79C70648-6AFF-569B-B45A-AF32532B9395 10.3897/BDJ.12.e130385.suppl5 Supplementary material 5 Pairwise genetic differentiation (Fst) between populations for *D.mun* species Data type Genomic File: oo_1068863.docx https://binary.pensoft.net/file/1068863 Thi Tuyet Xuan Bui, Dinh Duy Vu

1C6F7AE6-6F66-55D6-A0BB-CE46317E3DBB 10.3897/BDJ.12.e130385.suppl6 Supplementary material 6 Number of individuals for each population assigned. Each cluster was obtained from DAPC without prior information Data type Genomic File: oo_1068864.docx https://binary.pensoft.net/file/1068864 Thi Tuyet Xuan Bui, Dinh Duy Vu

5E44FEA0-BAEE-5EF6-88E4-9E80B6691575 10.3897/BDJ.12.e130385.suppl7 Supplementary material 7 Percentage of ancestry for three *D.mun* populations Data type Genomic File: oo_1068865.docx https://binary.pensoft.net/file/1068865 Thi Tuyet Xuan Bui, Dinh Duy Vu

## Figures and Tables

**Table 1.** Sampling location and genetic diversity within *D.mun* populations using 10 SSR loci.

Population code	Locations	Latitude (E)	Longitude (N)	*N*	*N_A_*	*N_E_*	*P*%	*Ho*	*He*	*F_IS_*	*F_IS_IIM*
**NH**	Na Hang Nature Reserve, Tuyen Quang Province	22°24'35"	105°25'11"	28	3.8	2.3	100	0.53	0.55	0.05	0.02
**NS**	Ngoc Son Nature Reserve, Hoa Binh Province	20°24'22"	105°20'14"	26	4	2.8	100	0.58	0.59	0.002	0.03
**CP**	Cuc Phuong National Park, Ninh Binh Province	20°20'16''	105°36'21''	28	4.1	2.9	100	0.57	0.6	0.034	0.01
**Species level**					3.9	2.8	100	0.56	0.58	0.029	0.02
**Note**: *N*: population size; *N_A_*: means number of alleles per locus; *N_E_*: means number of effective alleles; P%: percentage of polymorphic loci; *Ho* and *H_E_*: mean observed and expected heterozygosities, respectively; *F_IS_*: inbreeding coefficient; The individual inbreeding model was performed to evaluate the *F_IS_* index for null allele frequency *F_IS_IIM*.											

**Table 2.** Length distribution of assembly transcript and unigenes for *D.mun*.

**Length range (bp)**	**Unigene**	**Transcripts**
200-300	33,327 (36.57%)	37,823 (24%)
300-500	26,456 (29.03%)	32,955 (20.92%)
500-1000	17,051 (18.71%)	30,838 (19.57%)
1000-2000	9,067 (9.95%)	31,236 (19.83%)
>2000	5,233 (5.74%)	24,701 (15.68%)
Total Number	91,134	157,553
Total Length	58,649,736	166,642,297
N50 Length	957	1,867
Mean Length	645.55	105.77

**Table 3.** Functional annotation of *D.mun* in different databases.

**Annotated database**	**Annotated_No.**	**Percentage (%)**	**300–1000 (bp)**	≥ **1000 (bp)**
**COG**	9,666	10.61	3,022	4,821
**GO**	19,929	21.87	7,990	7,066
**KEGG**	12,017	13.19	4,721	4,878
**KOG**	18,499	20.30	7,399	7,326
**Pfam**	20,799	22.82	7,706	9,764
**Swissprot**	21,134	23.19	8,631	8,991
**eggNOG**	30,531	33.50	12,487	11,544
**Nr**	32,798	35.99	13,646	11,766
**All**	33,421	36.67	13,852	11,794

**Table 4.** Frequency of SSRs, based on repeat types in *D.mun* transcriptome.

**Number of repeat**	**Repeat type**						**Total**	**Percentage (%)**
	**Mono**-	**Di**-	**Tri**-	**Tetra**-	**Penta**-	**Hexa**-		
5	0	0	1056	75	13	6	1,150	12.25
6	0	633	535	10	2	4	1,184	12.61
7	0	394	251	0	1	0	646	6.88
8	0	409	21	0	0	0	430	4.58
9	0	462	0	0	1	0	463	4.93
10	1354	380	1	0	0	0	1,735	18.48
11	732	104	0	0	0	0	836	8.90
12	565	2	0	0	0	0	567	6.04
13	423	0	0	0	0	0	423	4.50
14	397	0	0	0	0	0	397	4.23
15	346	0	1	0	0	0	347	3.70
16	274	0	0	0	0	0	274	2.92
17	247	0	0	0	0	0	247	2.63
18	217	0	0	0	0	0	217	2.31
19	232	0	0	0	0	0	232	2.47
20	140	0	0	0	0	0	140	1.49
21	77	0	0	0	0	0	77	0.82
22	20	0	0	0	0	0	20	0.21
23	4	0	0	0	0	0	4	0.04
24	2	0	0	0	0	0	2	0.02
**Total**	**5,030**	**2,384**	**1,865**	**85**	**17**	**10**	**9,391**	
**Percentage (%)**	53.56	25.39	19.86	0.91	0.18	0.11		

**Table 5.** Genetic parameters in ten SSR loci for *D.mun*

**Locus**	**Sequence of primer (5'–3')**	**Motif type**	**Size (bp)**	***N_A_***	***N_E_***	**Null allele**	**PIC**	***H* o**	***H_E_***	***F_IS_***	***F_IT_***	***F_ST_***	***P*_HWE_**
**SSR01**	F: GAGGTGGATGAGGTGTTCGT R: GAGAAGGTTAGGGAGCCAGC	(GGA)_7_	251	5.00	2.66	No	0.86	0.66	0.61	-0.08	-0.03	0.05	***
**SSR 02**	F: TCCCACCAATCATCTTCTCC R: CACAGCAGAACCGCTTTGTA	(TC)_11_	193	4.00	3.02	No	0.75	0.85	0.66	-0.28	-0.26	0.02	ns
**SSR03**	F: TTTTTGGTTTCGGGACTTTG R: TCACCAAACAACAGGGTTCA	(TA)_11_	174	3.33	2.59	No	0.80	0.87	0.60	-0.45	-0.37	0.06	***
**SSR04**	F: AGCTTCATTCAAAGGCGCTA R: TCATTTCCCTTTGCCAAGAC	(CA)_11_	185	4.00	2.81	No	0.74	0.95	0.64	-0.48	-0.47	0,.0	***
**SSR05**	F: CATGTTCAAAGCCATTGTGG R: GCAATTGCAGCACCTCATTA	(GAG)10	265	3.33	2.43	No	0.76	0.25	0.58	0.58	0.60	0.06	ns
**SSR06**	F: GGAGCTATCCTCCCCTCAAC R: TCAGGCCATGAGACGTTGTA	(TTTC)_6_	273	4.00	2.14	No	0.72	0.34	0.53	0.37	0.37	0.01	ns
**SSR07**	F: TGAGGCTTCTTTGGATGCTT R: GTCTGGAATTTGGGGGAAAT	(CAT)_8_	216	3.00	2.35	No	0.62	0.76	0.57	-0.33	-0.28	0.04	**
**SSR08**	F: GCAACTTTGCATGTACCCCT R: AAGGCAAACAGGTTCCAATG	(AAAT)_6_	264	6.33	4.73	No	0.71	0.42	0.74	0.44	0.48	0.07	ns
**SSR09**	F: AGTGACGACAATAGCGGGTC R: TCTTTTGGTGGGGTTGAAAG	(CAGCAA)_5_	228	4.00	2.77	No	0.92	0.33	0.63	0.49	0.53	0.08	ns
**SSR10**	F: AATGAAAGACACCCACCCAG R: ATGAGACAGCCGTAATTGGG	(ACCAC)_9_	203	2.67	1.27	No	0.80	0.20	0.20	-0.02	0.03	0.05	***
**Mean**				**3.96**	**2.67**		**0.77**	**0.56**	**0.57**	**0.02**	**0.06**	**0.04**	
**Note**: *N_A_*: number of alleles; *N_E_*: effective alleles; *PIC*: polymorphism information content; *Ho* and *H_E_*: observed and expected heterozygosity; *F_IS_*: inbreeding coefficient, Null allele, the average null allele frequency, *F_IT_*: coefficient of total inbreeding; *F_ST_*: genetic differentiation index of Weir and Cockerham (1984), ns = not significant, *P < 0.05, **P < 0.01, ***P < 0.001													

**Table 6.** Analysis of molecular variance from natural populations for *D.mun* species produced.

	**df**	**Sum of squares**	**Variance components**	**Total** **variation (%)**	**Fixation indices**
Amongst populations	2	21.237	0.138	4	*F_IS_* = 0.046* F_ST_ = 0.045** F_IT_ = 0.089**
Amongst individuals within populations	79	243.537	0.136	4	*Nm* = 5.34
Within individuals	82	230.500	2.811	91	
Total	163	495,274	3.085	100	
**Note**: df, degree of freedom; *P < 0.05, ^∗∗^P < 0.01, ***p < 0.001					
