# Supplementary figures and images for: Population genetics analysis of Diospyrosmun A.Chev. ex Lecomte (Ebenaceae) based on EST-SSR markers derived from a novel transcriptome
Source: Biodivers Data J. 2024 Sep 18;12:e130385. doi: 10.3897/BDJ.12.e130385 (PMC11424986; doi:10.3897/BDJ.12.e130385)

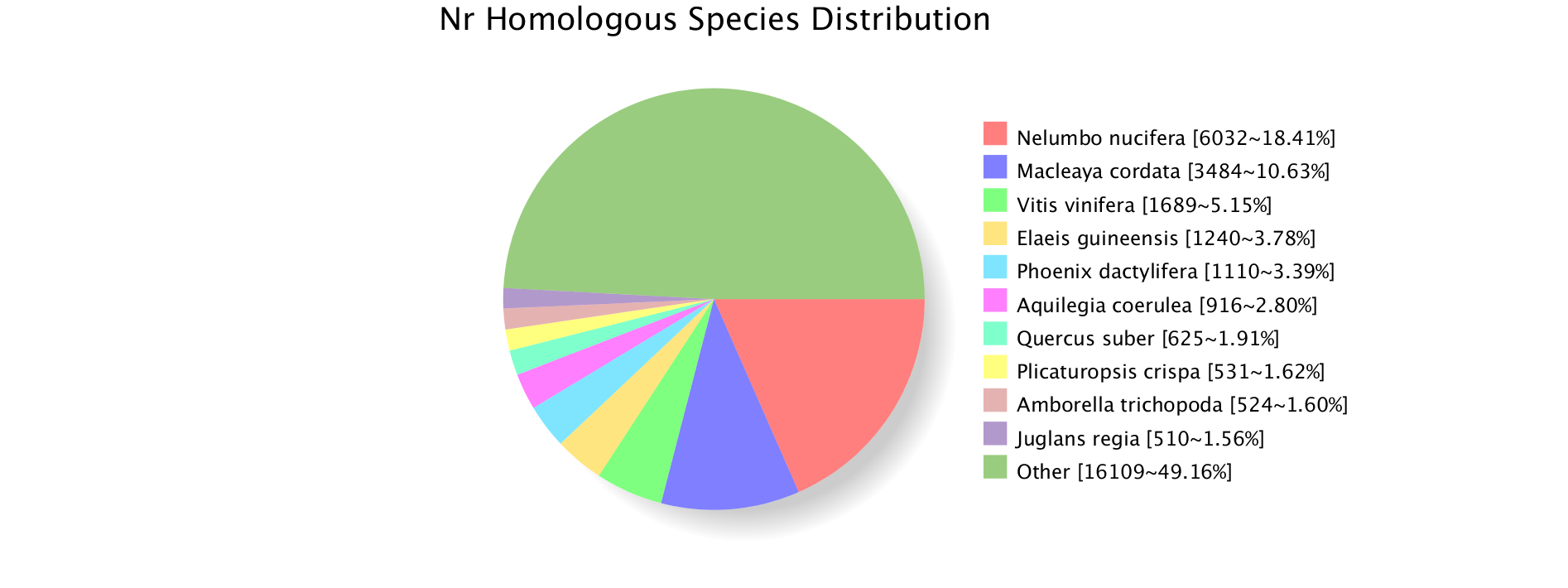

Supplement: Supplementary material 1 — Distribution of species search of unigenes against the Nr database. [file bdj-12-e130385-s001.png]

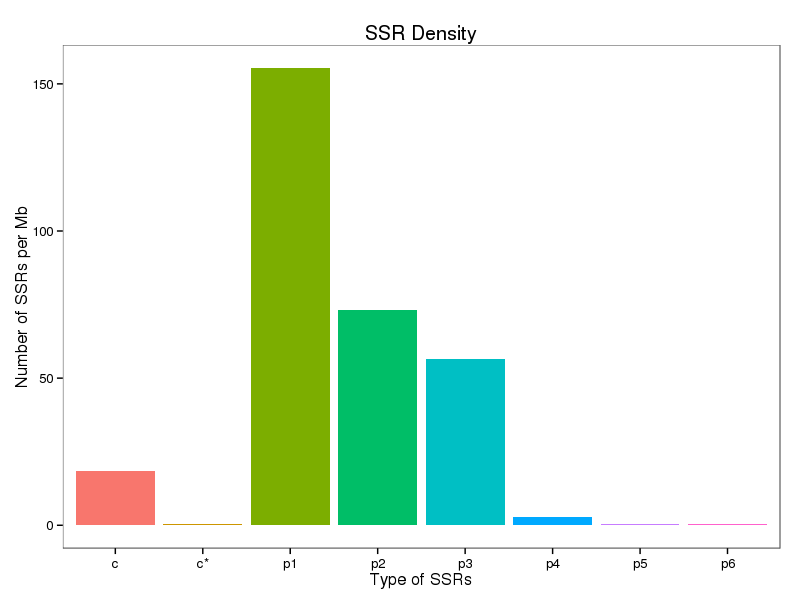

Supplement: Supplementary material 2 — Distribution type of EST-SSRs of D.mun [file bdj-12-e130385-s002.png]
