# Supplementary material for: Population genetics analysis of Diospyrosmun A.Chev. ex Lecomte (Ebenaceae) based on EST-SSR markers derived from a novel transcriptome
Source: Biodivers Data J. 2024 Sep 18;12:e130385. doi: 10.3897/BDJ.12.e130385 (PMC11424986; doi:10.3897/BDJ.12.e130385)
Supplement: Supplementary material 3 — Summary of analyses of expressed sequence Tag–Simple Sequence repeat (EST-SSRs) in D.mun [file bdj-12-e130385-s003.docx]

| **Item** | **Parameters** | **Number** |
| --- | --- | --- |
| EST-SSR | Total number of sequences examined | 14,300 |
|  | Total size of examined sequences (bp) | 28,647,926 |
|  | Total number of identified SSRs | 9,391 |
|  | Number of SSR containing sequences | 6,545 |
|  | Number of sequences containing more than 1 SSR | 2,035 |
|  | Number of SSRs present in compound formation | 586 |
