# Supplementary material for: Population genetics analysis of Diospyrosmun A.Chev. ex Lecomte (Ebenaceae) based on EST-SSR markers derived from a novel transcriptome
Source: Biodivers Data J. 2024 Sep 18;12:e130385. doi: 10.3897/BDJ.12.e130385 (PMC11424986; doi:10.3897/BDJ.12.e130385)
Supplement: Supplementary material 4 — Frequency distribution of SSRs based on motif types in D.mun transcriptome [file bdj-12-e130385-s004.docx]

| **Microsatellite motif** | **Number of repeats** | | | | | | | **Total** | **Percentage (%)** |
| --- | --- | --- | --- | --- | --- | --- | --- | --- | --- |
|  | **5** | **6** | **7** | **8** | **9** | **10** | **>10** |  |  |
| A/T | - | - | - | - | - | 1337 | 3644 | 4981 | 53.04 |
| C/G | - | - | - | - | - | 17 | 32 | 49 | 0.52 |
| AC/GT | - | 76 | 35 | 36 | 19 | 18 | 11 | 195 | 2.08 |
| AG/CT | - | 481 | 317 | 315 | 372 | 298 | 69 | 1852 | 19.72 |
| AT/AT | - | 72 | 41 | 58 | 71 | 64 | 26 | 332 | 3.54 |
| CG/CG | - | 4 | 1 |  |  |  |  | 5 | 0.05 |
| AAC/GTT | 44 | 20 | 23 | 2 |  |  | 1 | 90 | 0.96 |
| AAG/CTT | 377 | 244 | 97 | 2 |  |  |  | 720 | 7.67 |
| AAT/ATT | 61 | 38 | 20 | 1 |  |  |  | 120 | 1.28 |
| ACC/GGT | 48 | 27 | 12 | 2 |  |  |  | 89 | 0.95 |
| ACG/CGT | 28 | 6 | 4 | 1 |  |  |  | 39 | 0.42 |
| ACT/AGT | 6 | 4 | 2 | 1 |  |  |  | 13 | 0.14 |
| AGC/CTG | 162 | 57 | 29 | 4 |  |  |  | 252 | 2.68 |
| AGG/CCT | 139 | 71 | 32 | 3 |  | 1 |  | 246 | 2.62 |
| ATC/ATG | 146 | 57 | 22 | 2 |  |  |  | 227 | 2.42 |
| CCG/CGG | 45 | 11 | 10 | 3 |  |  |  | 69 | 0.73 |
| AAAC/GTTT | 6 | 1 |  |  |  |  |  | 7 | 0.07 |
| AAAG/CTTT | 18 | 2 |  |  |  |  |  | 20 | 0.21 |
| AAAT/ATTT | 20 | 2 |  |  |  |  |  | 22 | 0.23 |
| AACC/GGTT | 2 |  |  |  |  |  |  | 2 | 0.02 |
| AAGC/CTTG | 1 |  |  |  |  |  |  | 1 | 0.01 |
| AAGG/CCTT | 3 |  |  |  |  |  |  | 3 | 0.03 |
| AATC/ATTG | 5 | 1 |  |  |  |  |  | 6 | 0.06 |
| AATG/ATTC | 1 |  |  |  |  |  |  | 1 | 0.01 |
| ACAG/CTGT | 1 |  |  |  |  |  |  | 1 | 0.01 |
| ACAT/ATGT |  | 1 |  |  |  |  |  | 1 | 0.01 |
| ACCC/GGGT | 2 |  |  |  |  |  |  | 2 | 0.02 |
| AGAT/ATCT | 4 | 1 |  |  |  |  |  | 5 | 0.05 |
| AGCG/CGCT | 1 |  |  |  |  |  |  | 1 | 0.01 |
| AGGC/CCTG | 1 |  |  |  |  |  |  | 1 | 0.01 |
| AGGG/CCCT | 5 | 1 |  |  |  |  |  | 6 | 0.06 |
| ATCC/ATGG | 2 | 1 |  |  |  |  |  | 3 | 0.03 |
| ATCG/ATCG | 3 |  |  |  |  |  |  | 3 | 0.03 |
| AAATC/ATTTG | 1 |  |  |  |  |  |  | 1 | 0.01 |
| AAATG/ATTTC | 1 |  |  |  |  |  |  | 1 | 0.01 |
| AACCC/GGGTT |  | 1 |  |  |  |  |  | 1 | 0.01 |
| AACCG/CGGTT |  |  | 1 |  |  |  |  | 1 | 0.01 |
| AACTC/AGTTG | 1 |  |  |  |  |  |  | 1 | 0.01 |
| AAGAG/CTCTT | 1 |  |  |  |  |  |  | 1 | 0.01 |
| AAGGG/CCCTT | 1 |  |  |  |  |  |  | 1 | 0.01 |
| AATAT/ATATT | 1 |  |  |  |  |  |  | 1 | 0.01 |
| AATCG/ATTCG | 1 |  |  |  |  |  |  | 1 | 0.01 |
| AATTC/AATTG | 1 |  |  |  |  |  |  | 1 | 0,01 |
| ACACC/GGTGT |  |  |  |  | 1 |  |  | 1 | 0.01 |
| ACCAT/ATGGT |  | 1 |  |  |  |  |  | 1 | 0.01 |
| ACGAG/CGTCT | 1 |  |  |  |  |  |  | 1 | 0.01 |
| AGAGG/CCTCT | 1 |  |  |  |  |  |  | 1 | 0.01 |
| AGGGC/CCCTG | 1 |  |  |  |  |  |  | 1 | 0.01 |
| ATATC/ATATG | 1 |  |  |  |  |  |  | 1 | 0.01 |
| ATCCG/ATCGG | 1 |  |  |  |  |  |  | 1 | 0.01 |
| AACAGC/CTGTTG | 1 |  |  |  |  |  |  | 1 | 0.01 |
| AACTTC/AAGTTG | 1 |  |  |  |  |  |  | 1 | 0.01 |
| AAGCAG/CTGCTT | 1 |  |  |  |  |  |  | 1 | 0.01 |
| AATCCC/ATTGGG |  | 1 |  |  |  |  |  | 1 | 0.01 |
| AATGAG/ATTCTC | 1 |  |  |  |  |  |  | 1 | 0.01 |
| ACAGAT/ATCTGT |  | 1 |  |  |  |  |  | 1 | 0.01 |
| ACCGCC/CGGTGG | 1 |  |  |  |  |  |  | 1 | 0.01 |
| ACGATC/ATCGTG |  | 1 |  |  |  |  |  | 1 | 0.01 |
| AGAGGC/CCTCTG |  | 1 |  |  |  |  |  | 1 | 0.01 |
| AGCCCT/AGGGCT | 1 |  |  |  |  |  |  | 1 | 0.01 |
