# Supplementary material for: Population genetics analysis of Diospyrosmun A.Chev. ex Lecomte (Ebenaceae) based on EST-SSR markers derived from a novel transcriptome
Source: Biodivers Data J. 2024 Sep 18;12:e130385. doi: 10.3897/BDJ.12.e130385 (PMC11424986; doi:10.3897/BDJ.12.e130385)
Supplement: Supplementary material 5 — Pairwise genetic differentiation (Fst) between populations for D.mun species [file bdj-12-e130385-s005.docx]

|  | **NS** | **NH** | **CP** |  |
| --- | --- | --- | --- | --- |
| **NS** | 0.000 |  |  | **NS** |
| **NH** | 0.044*** | 0.000 |  | **NH** |
| **CP** | 0.009*** | 0.082** | 0.000 | **CP** |
|  | **NS** | **NH** | **CP** |  |
| *Note:* ***P<0.01, ***P<0.001* | | | | |
