# Supplementary material for: Population genetics analysis of Diospyrosmun A.Chev. ex Lecomte (Ebenaceae) based on EST-SSR markers derived from a novel transcriptome
Source: Biodivers Data J. 2024 Sep 18;12:e130385. doi: 10.3897/BDJ.12.e130385 (PMC11424986; doi:10.3897/BDJ.12.e130385)
Supplement: Supplementary material 6 — Number of individuals for each population assigned. Each cluster was obtained from DAPC without prior information [file bdj-12-e130385-s006.docx]

| **Population** | **Cluster 1** | **Cluster 2** | **Cluster 3** |
| --- | --- | --- | --- |
| **NS** | 14 | 1 | 11 |
| **NH** | 9 | 11 | 8 |
| **CP** | 7 | 14 | 7 |
