# Supplementary material for: Population genetics analysis of Diospyrosmun A.Chev. ex Lecomte (Ebenaceae) based on EST-SSR markers derived from a novel transcriptome
Source: Biodivers Data J. 2024 Sep 18;12:e130385. doi: 10.3897/BDJ.12.e130385 (PMC11424986; doi:10.3897/BDJ.12.e130385)
Supplement: Supplementary material 7 — Percentage of ancestry for three D.mun populations [file bdj-12-e130385-s007.docx]

| **Populations** | **Genetic group** | | |
| --- | --- | --- | --- |
|  | **I (orange)** | **II (blue)** | **III (purple)** |
| **NS** | 0.473 | 0.160 | 0.368 |
| **NH** | 0.294 | 0.379 | 0.327 |
| **CP** | 0.160 | 0.477 | 0.363 |
